# Relevance of silica surface morphology in Ampyra adsorption. Insights from quantum chemical calculations

**DOI:** 10.1039/c8ra08792j

**Published:** 2019-02-05

**Authors:** E. Noseda Grau, G. Román, A. Díaz Compañy, G. Brizuela, A. Juan, S. Simonetti

**Affiliations:** Instituto de Física del Sur (IFISUR), Departamento de Física, Universidad Nacional del Sur (UNS), CONICET Av. L. N. Alem 1253 B8000CPB – Bahía Blanca Argentina ssimonet@uns.edu.ar; Comisión de Investigaciones Científicas (CIC) Calle 526 e/10 y 11 1900 – La Plata Argentina; Universidad Tecnológica Nacional (UTN) 11 de Abril 461 B8000LMI – Bahía Blanca Argentina

## Abstract

Theoretical calculations are performed using the Vienna Ab-initio simulation package (VASP) to understand the mechanisms that control the adsorption of Ampyra drug on the different crystallographic planes of β-cristobalite: the hydroxylated (111) and (100) surfaces. The Ampyra-silica interaction is most favored on the (100) surface where the entire ring of the molecule interacts with the surface while on the (111) face, lesser exchange and fewer non-polar atoms are involved. Calculations show that the interactions mainly occur at the interface between the Ampyra and the closest silanol groups, according to the formation of the H-bonding interactions. The results indicate that the H-bonds have an important influence on the adsorption of the Ampyra. In consequence, adsorption on the (111) surface is observed to a lesser extent than on the (100) surface according the smaller hydroxyl density.

## Abstract 2

DFT calculations are performed to understand the mechanisms that control the adsorption of Ampyra drug on the different crystallographic planes of β-cristobalite: the hydroxylated (111) and (100) surfaces.

## Graphical abstract

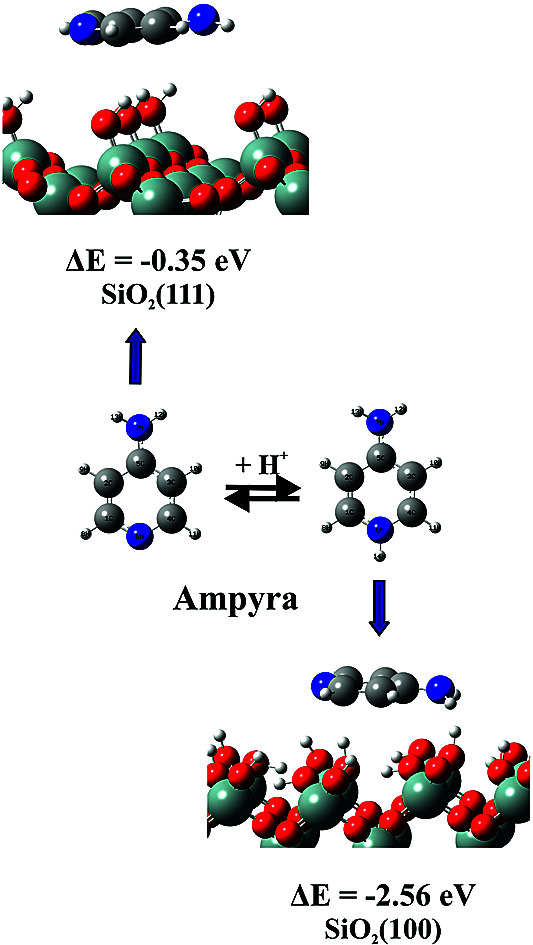

## 1. Introduction

Dalfampridine-ER (Ampyra), also known by its chemical name, 4-aminopyridine, or fampridine, was developed to maintain plasma levels of the drugs within a narrow therapeutic window were evaluated for their ability to improve multiple sclerosis (MS) being their most important symptom, disability when walking.^1–6^ Ampyra, a potassium channel blocker, is able to restore a nerve conduction block in demyelinated nerve fibers by prolonging the duration of the action potential.^7^ In some clinical trials in patients with multiple sclerosis, transient improvements in visual, oculomotor and motor functions were demonstrated after intravenous or oral administration of Ampyra. Therefore, it was approved by U.S. Food and Drug Administration (FDA) for the treatment of MS in 2010.^7^

In spite of the advance made with the introduction of new cytotoxic agents and medicine professional exercise, it is needed to explore the support of drugs on selected carrier matrixes for more effective pharmacological treatment. Treatment with drugs such as Ampyra has harmful side effects.^8^ Thus, it transport in delivery systems may help to reduce side effects and facilitates oral administration. Oral drug administration presents numerous advantages, including patient's tolerance and the lower costs related to drug preparation and dispensation.^9^ In addition, carrier may allow drugs to be released in a controlled manner preventing drug degradation.^10,11^

Silica surfaces have a key role in many applications dealing with molecule interactions, for instance, in chromatography,^12^ just to mention the most common one. Indeed, silica as stationary phase for liquid chromatography system is used in the pharmaceutical industry, in the analysis of contaminants, pesticides, bioanalytes, and drug residues in drinks and food samples, and in medical or environmental tests. Studies have shown that silica matrixes could improve drug delivery systems.^13–16^ Our group has previously studied the adsorption of molecules of industrial interest on silica surfaces using complemented computational and experimental techniques.^17,18^

**Fig. 1:**
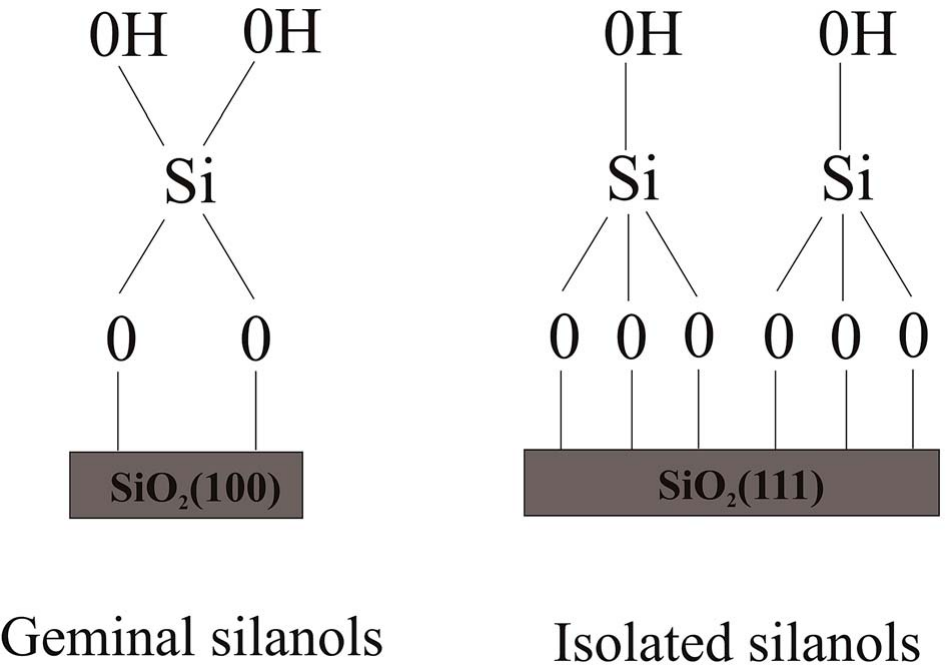
Silanol groups identified experimentally on the amorphous silica surfaces.

To design drug adsorbent, it is crucial to understand the adsorption mechanisms. The technological applications of silica were found to rely on its specific surface properties. Recently formed silica will include a distribution of reactive sites, which are known to fast react with atmospheric moisture, leading to the formation of surface silanol (Si–OH) groups. The concentration, distribution, and nature of silanols mainly decide the technologically significant properties of hydroxylated silica surface. The first step in the clarification of the reaction mechanism necessarily involves a detailed characterization of the surface silanols which are the guess reactive sites for the molecule adsorption. Although a complete microscopic description of the hydroxylated silica surface is still lacking, various experimental techniques including NMR,^19–21^ infrared^19,22–27^ and Raman^28,29^ spectroscopy, as well as chemical probes have provided many data on the properties of surface silanols which are often rationalized by modeling the surface as an alternation of patches of the hydroxylated (100) and (111) surfaces of β-cristobalite.^19,30^ This is the crystalline phase of silica with density and refractive index closest to those of amorphous silica.^31,32^ Expert suggested the existence of local ordering on amorphous silica surfaces.^33^ Moreover, the two main faces of β-cristobalite can sustain the two types of silanol groups identified experimentally on the amorphous silica surface, namely the “single” silanols (a single hydroxyl attached to a surface Si) typical of the (111) surface and the “geminal” silanols (two hydroxyls attached to the same surface Si), which are typical of the (100) surface (see Fig. 1).

The structure of a solid, its energetic heterogeneity, and surface chemical properties are the main factors influencing adsorption equilibria. A detailed atomistic knowledge of the adsorption mechanism of Ampyra on the hydroxylated (100) and (111) surfaces of β-cristobalite is obviously of paramount importance for the design of new, better performing coupling silica carriers and for the optimization of the technological process. Consequently, the computational study of the physicochemical characteristics of the surface is an element to explain and predict the strength of the drug–silica interactions to control the adsorption and release.

## 2. Computational and model

Density Functional Theory (DFT) calculations^34,35^ were performed for the study of structural and electronic system properties employing the projector-augmented wave (PAW) method^36–38^ as implemented in the Vienna Ab-initio Simulation Package (VASP).^39^ An energy cutoff of 500 eV was used to expand the Kohn–Sham orbitals into plane wave basis sets. The generalized gradient approximation (GGA) with the functional Perdew–Burke–Ernzerhof (PBE) were employed.^40,41^ The correction of Grimme-D2 was applied.^42^ A Monkhorst–Pack *k*-point mesh^43^ equivalent to 3 × 3 × 1 was taken for the full (reducible) Brillouin zone, allowing the convergence of total energy and forces.

The SiO_2_(111) and (100) surface models were obtained by bulk β-cristobalite, saturated with hydroxyl groups (Si–OH) and optimized by VASP calculations. The result is a silica surface whose silanol density is close to the experimental value for fully hydroxylated surface.^44^ The surface was represented with a periodically repeated slab containing five layers of atoms separated in the normal direction by a vacuum region; while a large box of (20 × 20 × 20) Å^3^ was used to obtain the molecular energy of the molecule. The Ampyra drug was placed on one side of the silica slab and its geometry was complete optimized together with the three upper layers. The system energy was found as the energy of the Ampyra molecule adsorbed on the silica surface ((100) or (111)) minus the sum of the free silica surface ((100) or (111)) and the free Ampyra molecule energies respectively where negative value indicates an exothermic process. The Density of States (DOS) and Bader charge exchange^45,46^ were also calculated.

## 3. Results and discussion

**Fig. 2:**
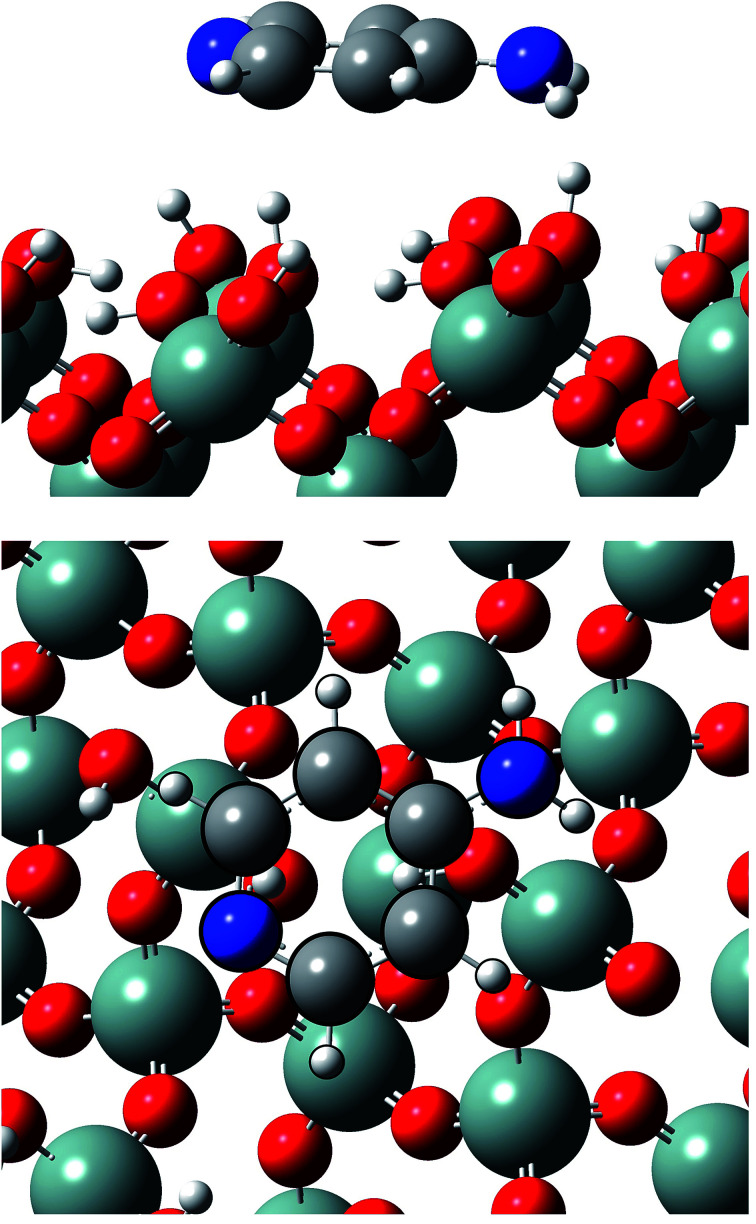
Lateral and top views of Ampyra adsorbed on SiO_2_(100) surface. Reference for atoms: silicon (green), oxygen (red), nitrogen (blue), carbon (grey) and hydrogen (white).

**Fig. 3:**
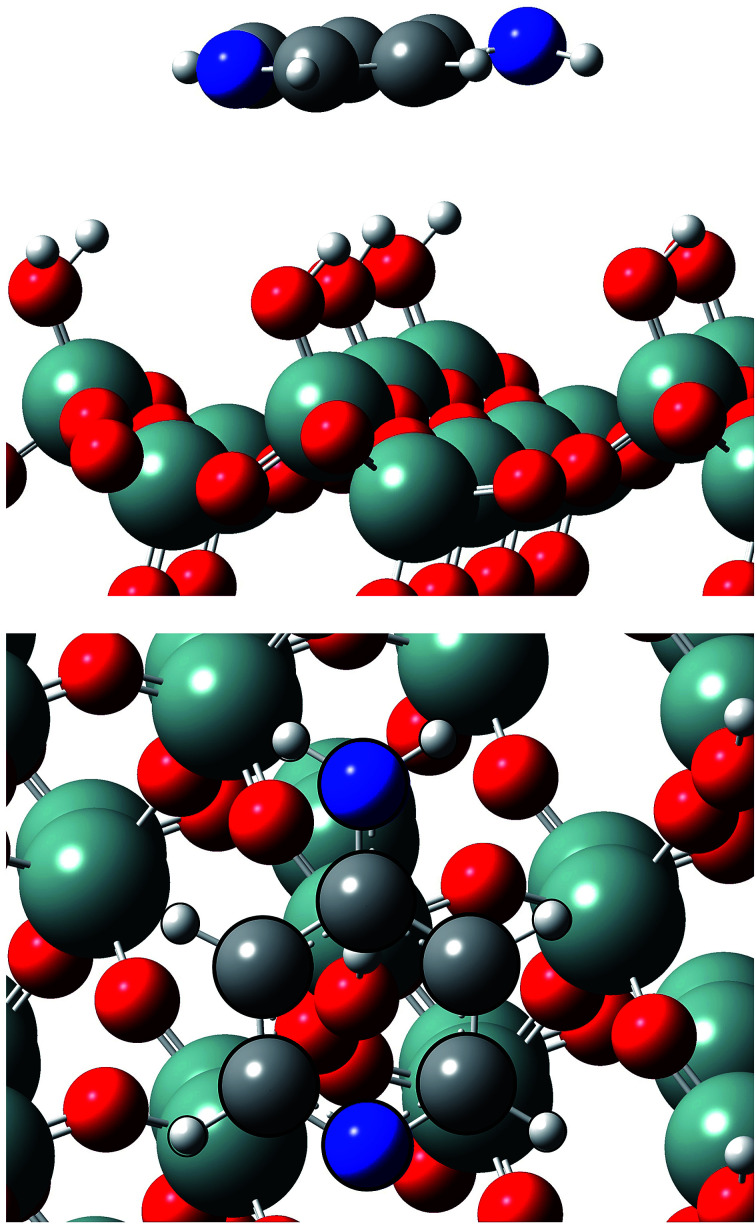
Lateral and top views of Ampyra adsorbed on SiO_2_(111) surface. Reference for atoms: silicon (green), oxygen (red), nitrogen (blue), carbon (grey) and hydrogen (white).

Calculations show that Ampyra molecule adsorbs nearly planar on both surfaces (see Fig. 2 and 3). When Ampyra absorbs on SiO_2_(100) surface is more stabilized than on SiO_2_(111). The Ampyra–SiO_2_(100) binding energy is −2.34 eV compare with the lesser Ampyra–SiO_2_(111) adsorption energy of −0.35 eV. Hydroxyl surface groups are H bonded with the molecule, serving as proton donor or proton-acceptor. Seven interactions of H-bonding type are formed in the Ampyra–SiO_2_(100) system (see Table 1); the H–O distances are between 2.80 Å and 3.33 Å (total of six H-bonding interactions where surface is proton-acceptor) and the N–H bonding distances is 2.25 Å (one H-bonding interaction where surface is proton-donor). On the other hand, only two H-bonding interactions are observed when Ampyra absorbs on the SiO_2_(111) surface (see Table 1); the N–H distances are 3.02 Å and 3.27 Å respectively and the surface is presented as proton-donor. β-cristobalite (100) hydroxylated face has a silanol density of 7.9 OH nm^−2^ while the (111) hydroxylated face has a silanol density of 4.5 OH nm^−2^.^34^ The surface hydroxyl density has important influence in the H-bonds formed between the drug and the surface. Conformity hydroxyl surface density, the smaller density is compatible to the fewer formed H-bonds and the lesser system stability. The adsorption energy does not necessarily proportional increase with the number of H-bonds there formed. Additional remarkable point is that H-bonding formed is a stronger interaction if a shorter H-bond length. On the other hand, no substantial drug structure modification is observed for the most favorable adsorption configurations; we note that the geometry of the adsorbed molecule looks quite similar to the isolated one.

**Table 1:** H-bond distances for Ampyra drug on SiO_2_(100) and SiO_2_(111) surfaces

H-bonds	Distances (Å)
**SiO**_**2**_ **(100)**	
9H–O_sup_	3.19
9H–O_sup_	3.24
11H–O_sup_	3.32
12H–O_sup_	3.33
13H–O_sup_	2.80
13H–O_sup_	3.17
6N–H_sup_	2.25

**SiO**_**2**_ **(111)**	
6N–H_sup_	3.02
7N–H_sup_	3.27

**Fig. 4:**
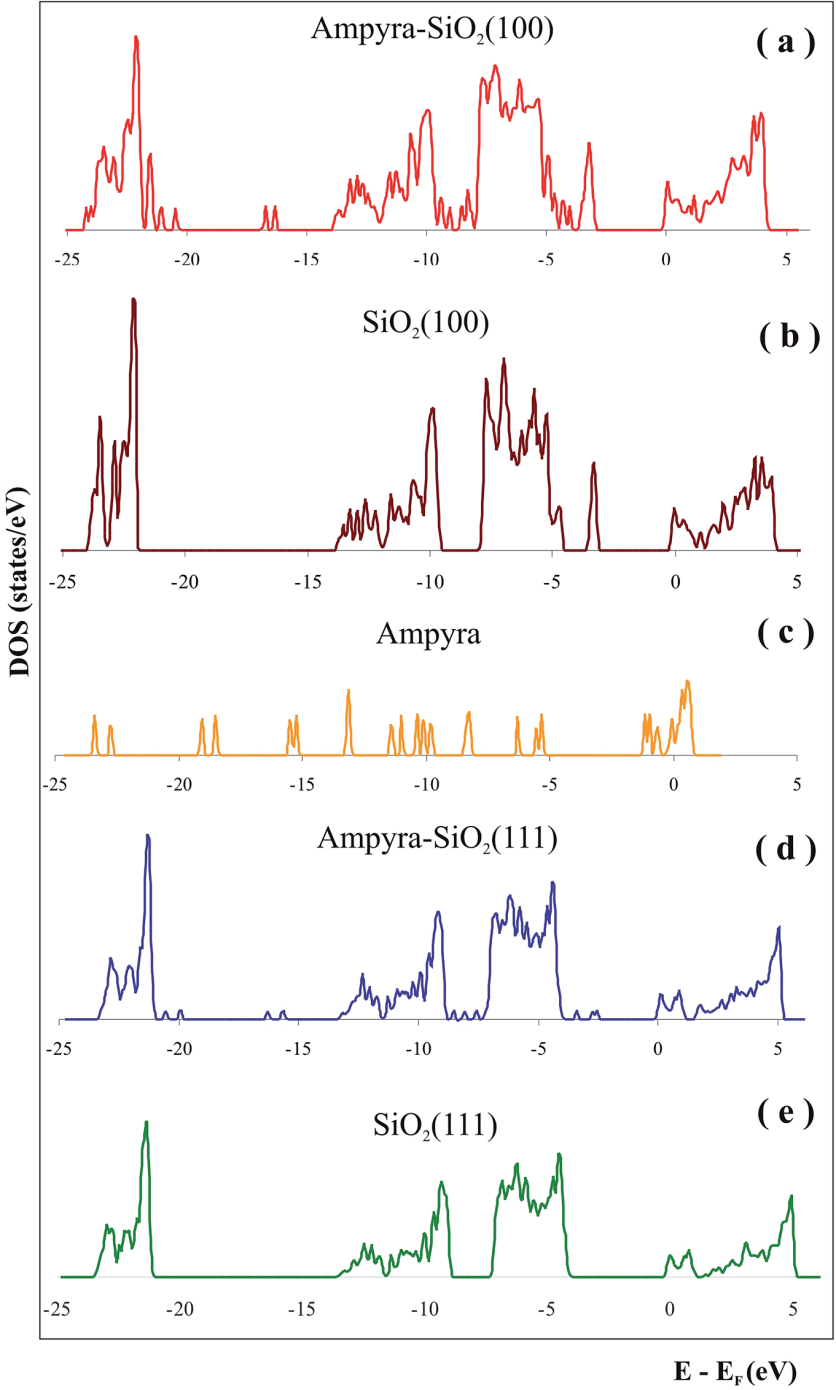
Density of states (DOS) of (a) Ampyra adsorbed on SiO_2_(100) surface, (b) isolated SiO_2_(100) surface, (c) isolated Ampyra, (d) Ampyra adsorbed on SiO_2_(111) surface and (e) isolated SiO_2_(111) surface.

We have made a state density (DOS) graphic of the system when the Ampyra is absorbed on the SiO_2_(111) hydrated surface (Fig. 4d). To compare the state densities, the DOS of both SiO_2_(111) isolated surface (Fig. 4e) and the isolated Ampyra drug (Fig. 4c) were also showed. There are bands associated with the interaction between Ampyra and the silica orbitals. The overlap is presented within −23 to −24 eV, −13 to −14 eV, −9 to −12 eV and −5 to −7 eV. Ampyra is presented with new states mainly in the part of the band in the range of −14 to −21 eV and −2 to −4 eV.

In general, when a drug contacts with the silica matrix, interactions by electrostatic forces of the partial charges produced by the movement of the electrons are established. The native silica in normal conditions has negative electrostatic charges with uniform zones of a great electron density. However, the drug molecule can be exclusively charged by positive charges, with deficit of electrons, negative, rich in electrons, or, the most usual, to exhibit zones of partial charge positive or negative in different locations of the molecule. It is understood that if the charges are of equal sign, they will be repulsed. Nevertheless, when electrostatic forces of different signs between the guest and the surface host are established, they must influence the drug retention in the silica matrix.

The calculations show that the electronic charge excharge happens mainly at the interface between the Ampyra atoms and hydroxyl groups closest to the molecule. When the Ampyra is adsorbed on the SiO_2_(111) surface, the molecule presents the charge changes showed in Table 2. The atoms most involved in the Ampyra–silica(111) interactions are 2C, 5C and 9H (see Table 2). This result shows that fewer local states of Ampyra are involved in the interaction with the (111) silica surface. The results show that there is a minor charge rearrangement in the Ampyra atoms and practically no changes are observed in the surface electronic structure.

**Table 2:** Partial charge on atoms for isolated and adsorbed Ampyra drug on SiO_2_(111) surface

Atom	Isolated Ampyra	Ampyra on SiO_2_(111)	Charge exchange
1C	2.6247	2.6151	0.0096
2C	3.9169	4.1668	−0.2499
3C	4.0750	4.0677	0.0073
4C	2.6321	2.6188	0.0133
5C	3.0690	2.8649	0.2041
6N	7.8148	7.8223	−0.0075
7N	7.9576	7.9633	−0.0057
8H	0.9466	0.9545	−0.0079
9H	1.0144	0.9605	0.0539
10H	0.9843	0.9678	0.0165
11H	0.9637	0.9665	−0.0028
12H	0.0002	0.0003	−0.0001
13H	0.0007	0.0004	0.0003

We have calculated the state density (DOS) of the system when the Ampyra is absorbed on the hydrated SiO_2_(100) surface (Fig. 4a). To compare the state densities, the DOS of the both isolated SiO_2_(100) surface (Fig. 4b) and Ampyra molecule (Fig. 4c) were also presented. The molecule-surface overlap is produced within −23 to −24 eV, −10 to −14 eV and −5 to −7 eV. The Ampyra exhibits new states mainly in the part of the band in the range of −16 to −22 eV and −8 to −9 eV. In general the mainly overlapping come from −5 eV to −14 eV where O p orbitals of silica surface better interacts with H atoms of Ampyra and this is in agreement with the formation of the H-bonding interactions.

The calculations show that the electronic charges mainly happen at the interface between the ring of Ampyra and hydroxyl groups closest to the molecule. When Ampyra is adsorbed on the SiO_2_(100) surface, the molecule present the charge changes presented in the Table 3. The atoms most involved in the Ampyra–silica(100) interaction are 2C, 3C, 4C, 5C, 6N, 9H and 10H (see Table 3). After adsorption, it is present a rearrangement of the electronic charge in the molecule; in consequence, changes in partial charge occur. This result shows that more atoms of Ampyra are involved in the interactions and it is in agreement with the most favorable adsorption energy obtained for the Ampyra–silica(100) system. On the other hand, the superficial silica atoms near to the molecule experiment changes in their electronic structure confirming the stronger drug–surface(100) interactions.

**Table 3:** Partial charge on atoms for isolated and adsorbed Ampyra drug on SiO_2_(100) surface

Atom	Isolated Ampyra	Ampyra on SiO_2_(100)	Charge exchange
1C	2.6247	2.6321	−0.0074
2C	3.9169	4.0770	−0.1601
3C	4.0750	3.9044	0.1706
4C	2.6321	2.8324	−0.2003
5C	3.0690	2.8575	0.2115
6N	7.8148	7.8399	−0.0251
7N	7.9576	7.9512	0.0064
8H	0.9466	0.9443	0.0023
9H	1.0144	0.9610	0.0534
10H	0.9843	1.0221	−0.0378
11H	0.9637	0.9476	0.0161
12H	0.0002	0.0006	−0.0004
13H	0.0007	0.0001	0.0006

The greatly noticeable charge for the adsorbed molecule on SiO_2_(100) surface confirms once again that the molecule-surface interaction is strengthened upon adsorption. For the H-bonds for adsorbed molecule on SiO_2_(111) surface, the charge rearrangement is less significant confirming the weaker interaction.

Much more enhanced H bonding in the adsorbed Ampyra molecule is observed on cristobalite (100) than on (111) face, which can be indicated from more number of H-bonds, some H bond lengths more shortened, and the redistribution of the charge in the molecule more noticeable. The Ampyra adsorption is highly dependent on the surface structure of the substrate. Its formation is mainly determined by the requirement of saturating hydrogen bonds among the molecule. The cristobalite (100) surface with geminal hydroxyls, which provide active sites by either H donating or accepting bonds, better satisfies this requirement.

**Fig. 5:**
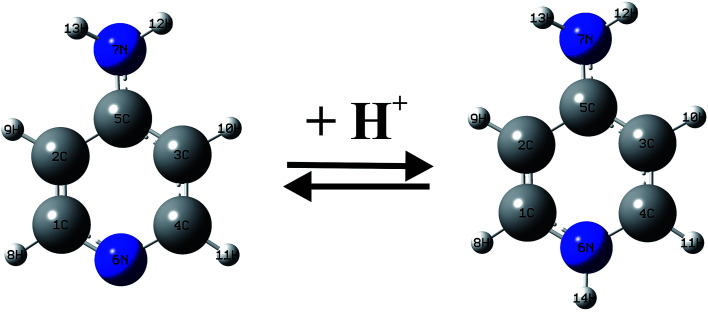
The equilibrium of Ampyra in aqueous solution.

Between 1990–2001 years, there have been a large number of studies of the Ampyra binding effect on native or mutated Kv channels that revealed the mechanisms of Ampyra blockage. For instance, it was noted that Ampyra blocks the Kv channel only in its protonated, cationic form (see Fig. 5).^47^ Also, some of the conclusions were about the probable binding site, and how Ampyra promotes the blockage.^48^ The real advance came only after the structure of the Kv channel was revealed giving important conclusions regarding aminopyridine binding.^49–51^ The previous findings that aminopyridines bind to the Kv channels in a protonated, cationic form is theoretically supported and explained. Further, it was suggested that the pyridine ring plays an active role in the interaction with the receptor site. This interaction with the protonated pyridine nitrogen can involve a cation-π interaction or a donor hydrogen bond. In fact, the pyridine ring was recognized as a pharmacophor, while a second amine group, at different relative positions of the pyridine nitrogen, can form one or more hydrogen bonds due to the C4 symmetry of the inner part of the pore in the Kv channel.

**Fig. 6:**
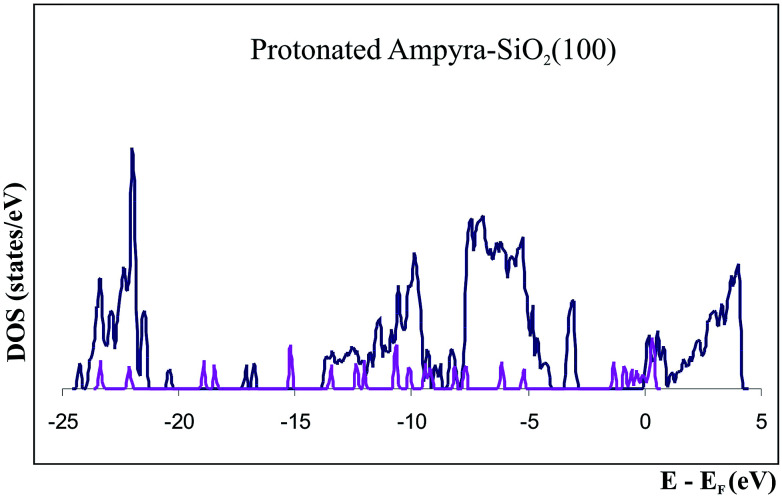
Density of states (DOS) of protonated Ampyra, isolated (pink) and adsorbed on SiO_2_(100) surface (blue).

We have studied the protonated specie of Ampyra adsorbed on SiO_2_(100) surface. The state density (DOS) of the system when the protonated Ampyra is absorbed on the hydrated SiO_2_(100) surface can be seen in Fig. 6. To compare the state densities, the DOS of the isolated protonated Ampyra molecule is also presented. The system is thermodynamically stable. The minimum energy is −2.56 eV and slightly stronger to that of neutral Ampyra adsorbed on the SiO_2_(100) surface (Δ*E* = −2.34 eV). After adsorption, all atoms of Ampyra molecule suffer electronic charge modifications. Bader charge analysis shows that the most important changes are reported on 1C and 2C atoms and significant changes are also reported on the others carbon of the molecule ring (see Table 4). The hydrogens bonded to the carbon atoms also present notable changes. An electron charge is transfer from the protonated nitrogen (6N) while electron charge is transfer to the hydrogen bonded to it (14H). Changes are also present in 9H and 10H atoms. Shojaie and Dehghan have reported that HOMO is almost distributed throughout the entire neutral Ampyra molecule, while the LUMO has an anti-bonding character and it is strongly distributed across this molecule with the exception of the NH_2_ group.^52^ In our work, calculations show that NH_2_ group presents notable changes in the 7N partial charge (2.4 times that of 6N) when protonated Ampyra is adsorbed on the SiO_2_(100) surface (see Table 4).

**Table 4:** Partial charge on atoms for isolated and adsorbed protonated Ampyra drug on SiO_2_(100) surface

Atom	Isolated protonated Ampyra	Protonated Ampyra on SiO_2_(100)	Charge exchange
1C	3.2585	4.5379	−1.2794
2C	3.9608	2.4924	1.4684
3C	3.9001	3.9622	−0.0621
4C	3.3136	3.2734	0.0402
5C	2.9382	2.9724	−0.0342
6N	7.8026	7.7951	0.0075
7N	7.9623	7.9801	−0.0178
8H	0.9615	0.9500	0.0115
9H	0.9556	0.9152	0.0404
10H	0.9818	0.9427	0.0391
11H	0.9576	0.9680	−0.0104
12H	0.0003	0.0006	−0.0003
13H	0.0008	0.0001	0.0007
14H	0.0003	0.0007	−0.0004

## 4. Conclusions

We have studied the interaction between Ampyra drug and the hydroxylated cristobalite (100) and (111) surfaces using the density-functional total-energy calculations within the generalized gradient approximation. These two single-crystal surfaces can support the two surface silanol configurations observed in experiment: the geminal and single silanols, and hence, represent prototype silica surface domains for general understanding of drug–silica interactions.

Our DFT calculations helped to understand the mechanisms and characteristics of the interactions that arise between the Ampyra drug and the two surfaces of silica: SiO_2_(111) and SiO_2_(100). The adsorption on silica in the different crystallographic planes presents differences due to the interaction of the Ampyra that is associated with the exposure of the silanol groups of the silica. For the Ampyra–silica interaction, charge exchanges were observed in the Ampyra on both surfaces, but the most favored to the adsorption is presented on the (100) surface where the most involved in the adsorption was the entire ring of Ampyra molecule, while on the (111) surface, lesser exchange and fewer non-polar atoms are involved. Calculations show that the stabilization of the electronic states of the Ampyra molecule on silica and the charge excharge, mainly occur at the interface between the Ampyra and the closest silanol groups, according to the formation of the H-bonding interactions. The results indicate that the H-bonds have an important influence on the adsorption of the Ampyra. In consequence, adsorption on the (111) surface is observed to a lesser extent than on the (100) surface according the smaller hydroxyl density.

## Conflicts of interest

There are no conflicts of interest to declare.

## Supplementary Material
